# Supplementary material for: Novel Driver Strength Index highlights important cancer genes in TCGA PanCanAtlas patients
Source: PeerJ. 2022 Aug 11;10:e13860. doi: 10.7717/peerj.13860 (PMC9375969; doi:10.7717/peerj.13860)
Supplement: Supplemental Information 2 [file peerj-10-13860-s002.zip › COHORTS/genes plots/stage/2021_8_16_14_9_distribution_stage_males_I.pdf]

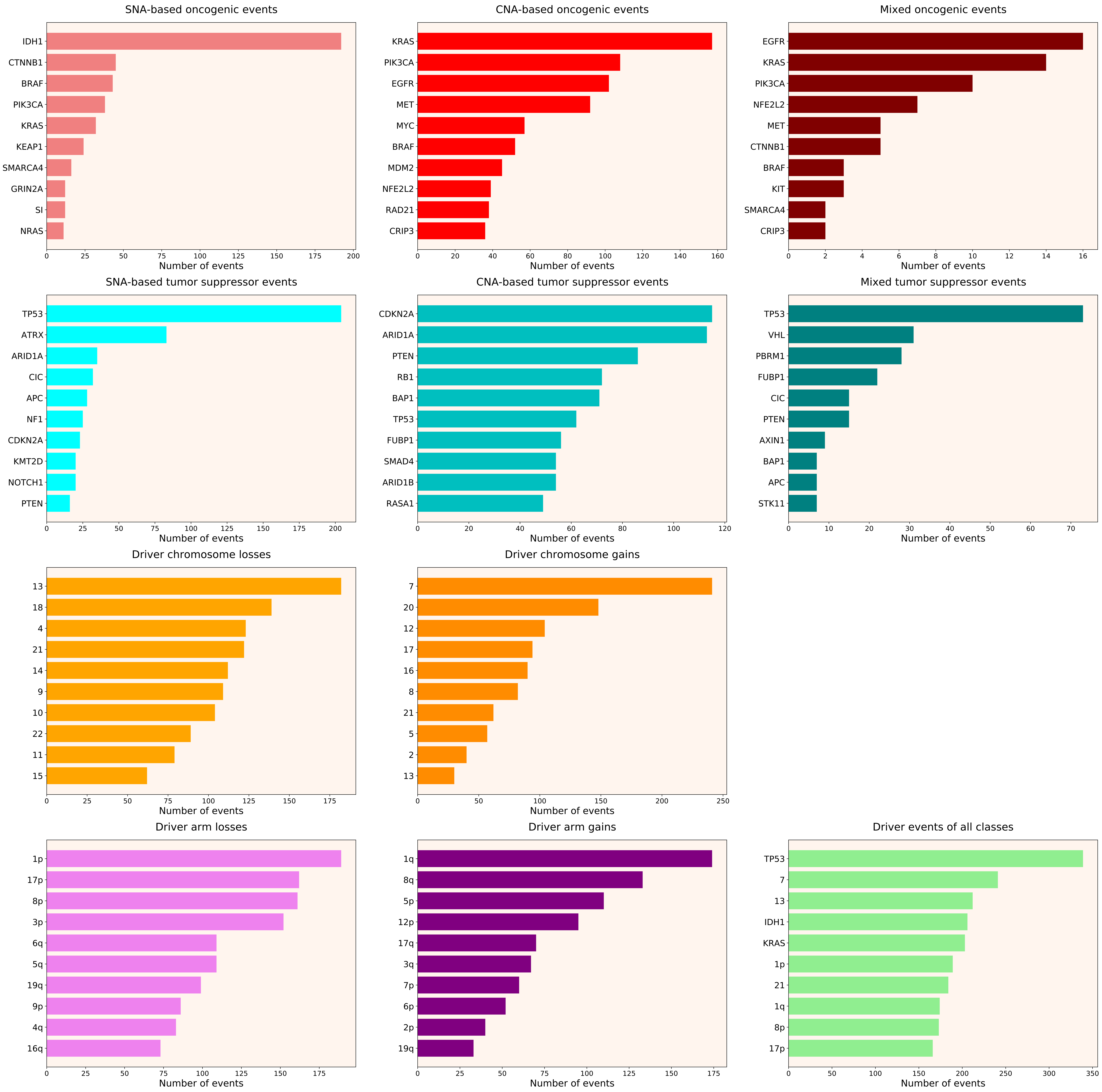
SNA-based oncogenic events
IDH1
CTNNB1
BRAF
PIK3CA
KRAS
KEAP1
SMARCA4
GRIN2A
SI
NRAS
0 25 50 75 100 125 150 175 200
Number of events
CNA-based oncogenic events
KRAS
PIK3CA
EGFR
MET
MYC
BRAF
MDM2
NFE2L2
RAD21
CRIP3
0 20 40 60 80 100 120 140 160
Number of events
Mixed oncogenic events
EGFR
KRAS
PIK3CA
NFE2L2
MET
CTNNB1
BRAF
KIT
SMARCA4
CRIP3
0 2 4 6 8 10 12 14 16
Number of events
SNA-based tumor suppressor events
TP53
ATRX
ARID1A
CIC
APC
NF1
CDKN2A
KMT2D
NOTCH1
PTEN
0 25 50 75 100 125 150 175 200
Number of events
CNA-based tumor suppressor events
CDKN2A
ARID1A
PTEN
RB1
BAP1
TP53
FUBP1
SMAD4
ARID1B
RASA1
0 20 40 60 80 100 120
Number of events
Mixed tumor suppressor events
TP53
VHL
PBRM1
FUBP1
CIC
PTEN
AXIN1
BAP1
APC
STK11
0 10 20 30 40 50 60 70
Number of events
Driver chromosome losses
13
18
4
21
14
9
10
22
11
15
0 25 50 75 100 125 150 175
Number of events
Driver chromosome gains
7
20
12
17
16
8
21
5
2
13
0 50 100 150 200 250
Number of events
Driver arm losses
1p
17p
8p
3p
6q
5q
19q
9p
4q
16q
0 25 50 75 100 125 150 175
Number of events
Driver arm gains
1q
8q
5p
12p
17q
3q
7p
6p
2p
19q
0 25 50 75 100 125 150 175
Number of events
Driver events of all classes
TP53
7
13
IDH1
KRAS
1p
21
1q
8p
17p
0 50 100 150 200 250 300 350
Number of events
